# Supplementary material for: The Effects of Filter Ventilation and Expanded Tobacco on the Tar, Nicotine and Carbon Monoxide Yields from Cigarettes Sold in Australia
Source: Int J Environ Res Public Health. 2024 Dec 31;22(1):50. doi: 10.3390/ijerph22010050 (PMC11764558; doi:10.3390/ijerph22010050)
Supplement: Supplementary file 1 [file ijerph-22-00050-s001.zip › ijerph-3346730-supplementary.pdf]

The effects of filter ventilation and expanded tobacco on the tar, nicotine and carbon monoxide yields from cigarettes sold in Australia.

Supplementary Information

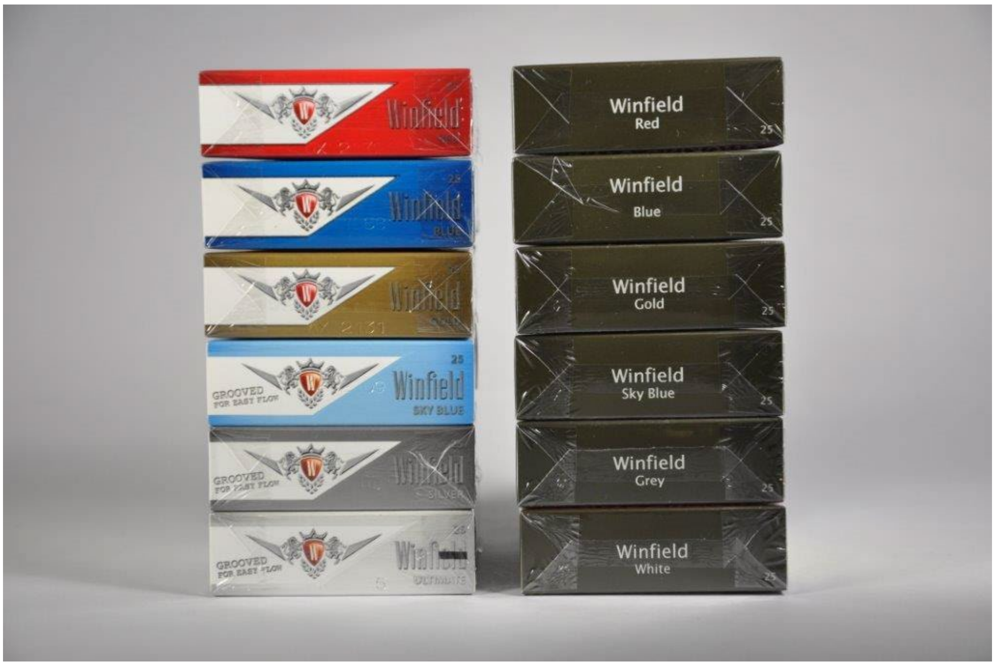

Figure S1. Examples of cigarette packets sold in Australia before (left) and after (right) the 2012 implementation of plain packaging legislation. The colors of the older packets are reflected in the brand variant names of the newer packets: red, blue, gold, sky blue, grey and white.

Table S1. Company and country of origin of cigarette brand variants sourced from major supermarkets and tobacconists in Australia, from February to April 2023.

Brand variant	Company[1]	Country of origin[2]
Bond street classic gold	PMI	Indonesia
Bond street classic blue	PMI	Indonesia
Bond street classic red	PMI	Indonesia
Chesterfield gold	PMI	Indonesia
Chesterfield blue	PMI	Indonesia
Chesterfield red	PMI	Indonesia
Derby gold	BATA	Singapore
Derby blue	BATA	Singapore
Derby red	BATA	Singapore
Holiday sun gold	BATA	Singapore
Holiday bright blue	BATA	Singapore
Holiday rich red	BATA	Singapore
Horizon orange	Imperial Brands	Taiwan or New Zealand
Horizon blue	Imperial Brands	Taiwan or New Zealand
Horizon red	Imperial Brands	Taiwan or New Zealand
Reef treasure gold	Richland Express	Luxembourg

Reef lagoon blue	Richland Express	Luxembourg
Reef coral red	Richland Express	Luxembourg
Rothmans gold	BATA	Singapore
Rothmans blue	BATA	Singapore
Rothmans red	BATA	Singapore
Winfield original gold	BATA	Singapore
Winfield original blue	BATA	Singapore
Winfield original red	BATA	Singapore

[1] BATA: British American Tobacco Australia, PMI: Philip Morris International
[2] According to package labels.

Table S2. Microscope photographs of filter ventilation holes in tipping paper.

**Brand variant**	**Color group**	**Photo**	**Filter ventilation (percent)**
Brand A	Gold	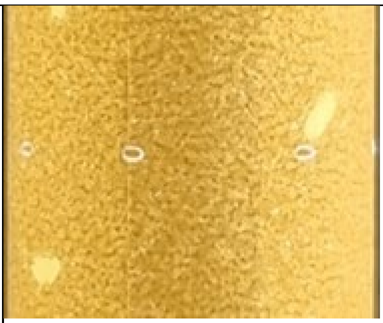	39.0
Brand B	Gold	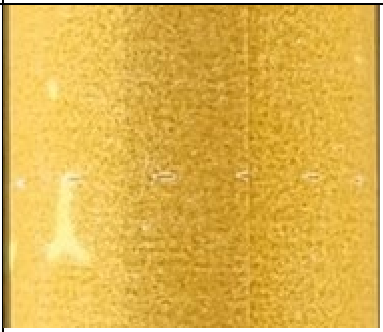	37.3
Brand C	Gold	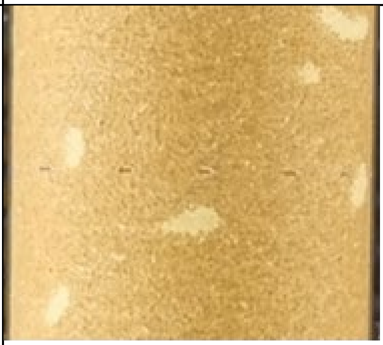	33.6

Brand D	Gold	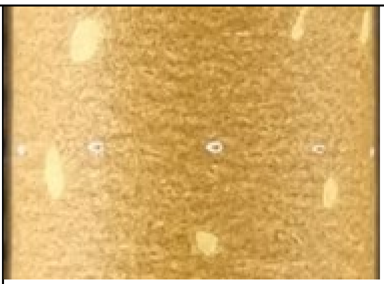	18.7
Brand E	Gold	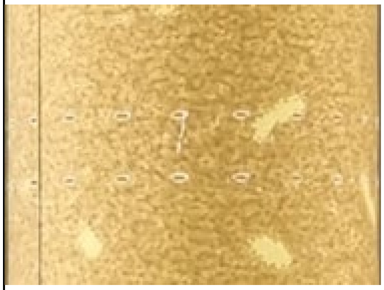	56.8
Brand F	Gold	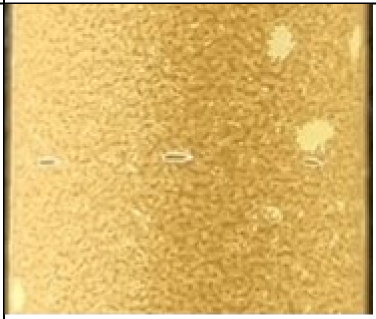	37.8
Brand G	Gold	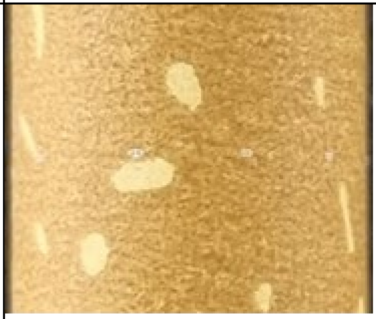	18.7
Brand H	Gold	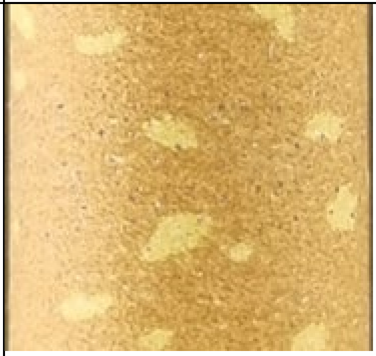	28.5

Brand A	Blue	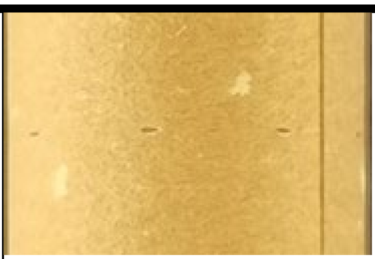	29.7
Brand B	Blue	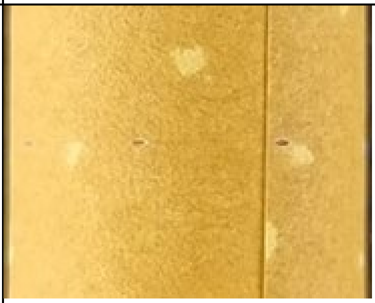	16.1
Brand C	Blue	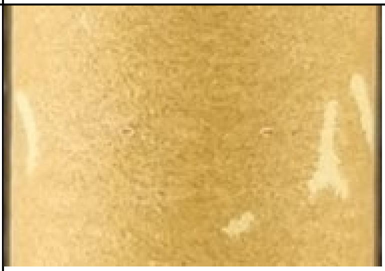	13.9
Brand D	Blue	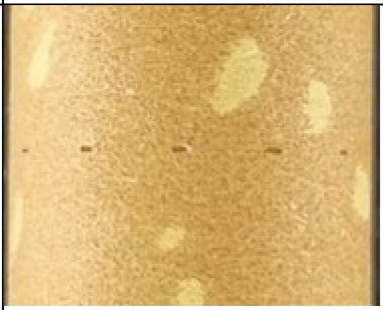	33.7
Brand E	Blue	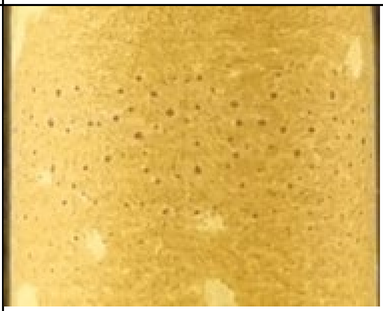	41.1
Brand F	Blue	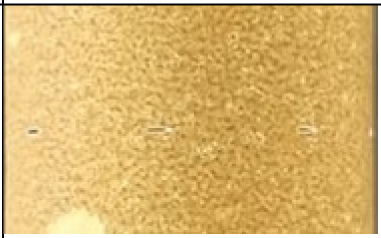	28.6

Brand G	Blue	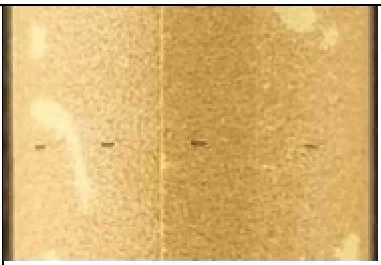	31.7
Brand H	Blue	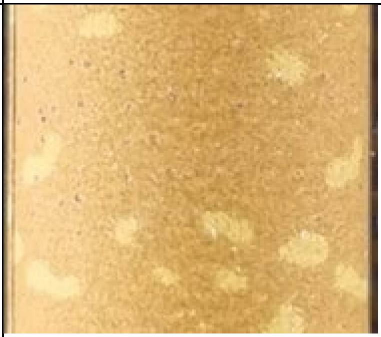	31.1
Brand A	Red	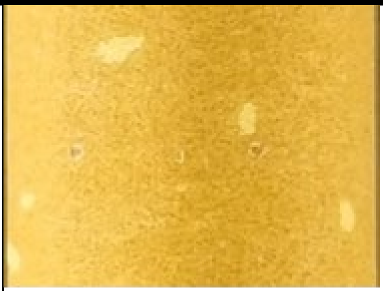	14.8
Brand B	Red	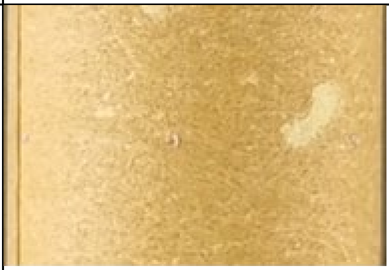	8.3
Brand C	Red	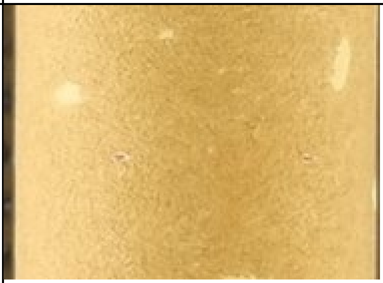	10.0

Brand D	Red	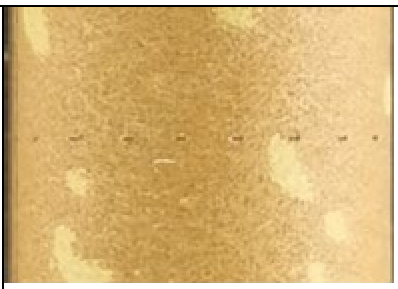	25.2
Brand E	Red	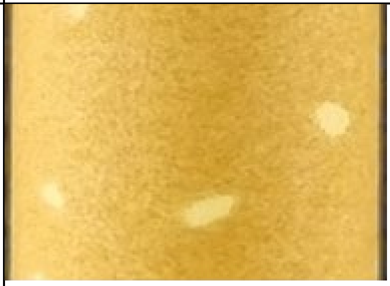	0.1
Brand F	Red	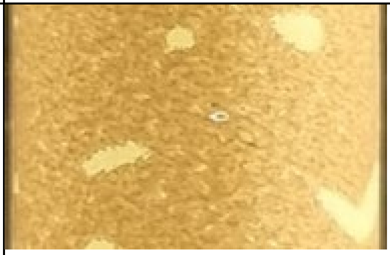	5.9
Brand G	Red	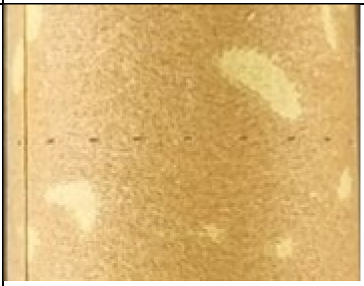	24.4
Brand H	Red	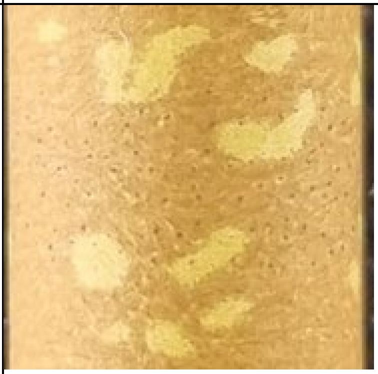	40.1

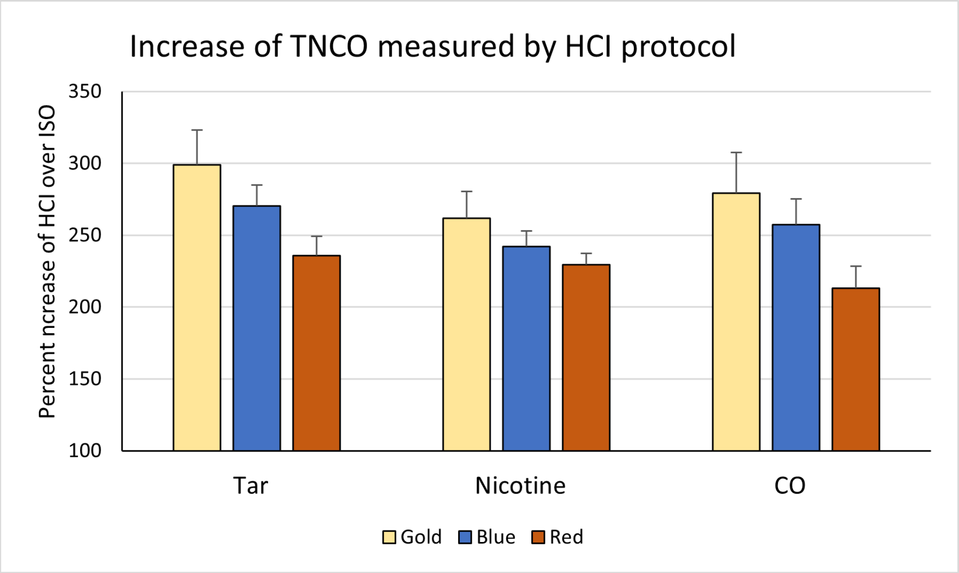

Figure S2. Mean and standard deviation (*n*=8 per group) of the percent difference between tar, nicotine and CO when measured using the HCI (Health Canada Intensive) versus the ISO (International Organization for Standardization) protocol.

Table S3. Mean proportion of product that is tobacco by weight, as calculated from the voluntary ingredient disclosures from BATA, PMI and Imperial Brands to the Australian Government in the year 2020.

**Tobacco company**	**Mean tobacco proportion of product[3]**		
	**Gold[4]**	**Blue[4]**	**Red[4]**
BATA[1]	71.3%	72.9%	73.9%
PMI[2]	73.4%	74.5%	75.45%
Imperial brands	73.8%	76.3%	76.3%

[1] BATA: British American Tobacco Australia
[2] PMI: Philip Morris International
[3] Tobacco proportion of products: tobacco weight divided by total product weight, expressed as percentage.
[4] Brand variants were selected for Gold (gold, rich gold, refined gold, yellow, amber or orange, but not menthol yellow) Blue (blue, rich blue, navy or bleue, but not sky blue) and Red (red or rich red). Products with crushable filters were not included.

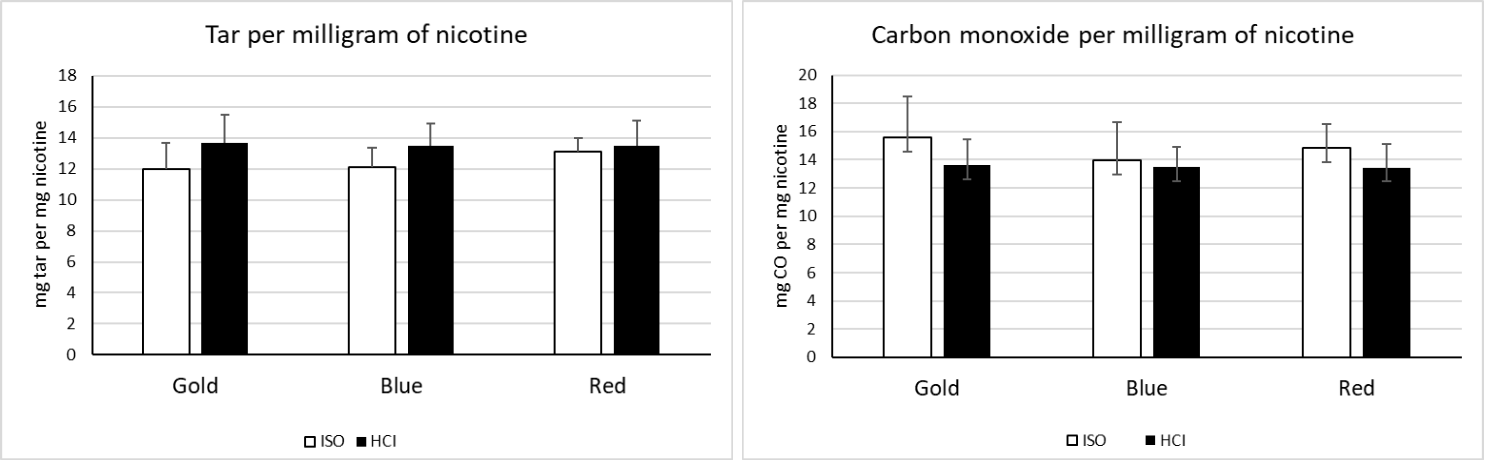

Figure S3. Mean and standard deviation (*n*=8 per group) of the tar and carbon monoxide (CO) produced in smoke when measured using the HCI (Health Canada Intensive) versus the ISO (International Organization for Standardization) protocol. Data are expressed as milligrams of tar or carbon monoxide per milligrams of nicotine per cigarette.
